# Saddle embolus and short-term outcomes in patients with intermediate-high-risk pulmonary embolism: a post hoc analysis of a randomized trial

**DOI:** 10.1016/j.rpth.2026.106921

**Published:** 2026-08-27

**Authors:** Frederik F. Lau, Emilie Sonne-Holm, Lia E. Bang, Jørn Carlsen, Peter Sommer Ulriksen, Samir Jawad, Pernille Palm, Connie Søe, Ekim Seven, Matias Greve Lindholm, Søren Fanø, Kristian Eskesen, Ole P. Kristiansen, Jesper Kjærgaard

**Affiliations:** 1 Department of Cardiology, The Heart Centre, Copenhagen University Hospital Rigshospitalet, Copenhagen, Denmark; 2 Department of Clinical Medicine, Faculty of Health and Medical Sciences, University of Copenhagen, Copenhagen, Denmark; 3 Department of Radiology, Copenhagen University Hospital Rigshospitalet, Copenhagen, Denmark; 4 Department of Cardiology, Copenhagen University Hospital Amager and Hvidovre, Copenhagen, Denmark; 5 Department of Cardiology, Zealand University Hospital, Roskilde, Denmark; 6 Department of Cardiology, Copenhagen University Hospital Herlev, Copenhagen, Denmark; 7 Department of Cardiology, Copenhagen University Hospital Gentofte, Copenhagen, Denmark; 8 Department of Cardiology, Copenhagen University Hospital Frederiksberg and Bispebjerg, Copenhagen, Denmark

**Keywords:** computed tomography angiography, pulmonary embolism, risk assessment, thrombolytic therapy

## Abstract

**Background:**

In patients with pulmonary embolism, a saddle-shaped embolus is perceived to be associated with disease severity. Although frequently observed in patients with acute intermediate-high-risk pulmonary embolism, its prognostic significance remains unclear.

**Objectives:**

In this study, we assessed the association between saddle embolism and short-term adverse outcomes in this risk subgroup.

**Methods:**

We conducted a post hoc analysis of a multicenter randomized clinical trial of 210 patients with intermediate-high-risk pulmonary embolism randomized to anticoagulation, systemic low-dose thrombolysis, or ultrasound-assisted catheter-directed low-dose thrombolysis. Saddle embolism was identified on baseline computed tomography pulmonary angiography. The primary outcome of this substudy was a composite of in-hospital mortality and/or the need for rescue thrombolysis. Secondary outcomes included saddle embolus resolution on follow-up imaging (after 48-96 hours) and other clinical outcomes.

**Results:**

Saddle embolism was present in 58 patients (28%). There were no major differences in demographic or clinical characteristics between patients with and without saddle embolism. Saddle embolism was not associated with the primary composite outcome (adjusted odds ratio, 0.87; 95% CI, 0.16-3.4; *P* = .85) or other clinical outcomes compared with no saddle embolism. However, ultrasound-assisted catheter-directed thrombolysis was associated with higher odds of saddle embolus resolution than heparin alone (odds ratio, 5.5; 95% CI, 1.06-35).

**Conclusion:**

In patients with intermediate-high-risk pulmonary embolism, we did not observe an association between saddle embolism and in-hospital mortality and/or the need for rescue thrombolysis. Ultrasound-assisted catheter-directed thrombolysis plus heparin was associated with a higher rate of short-term saddle embolus resolution than heparin alone.

## Essentials

• A saddle-shaped blood clot in the main lung artery is often considered especially dangerous.
• We studied 210 higher-risk patients treated in a clinical trial for blood clots in the lungs.
• Saddle-shaped clots were not linked to more early deaths or clinical deterioration.
• Saddle-shaped clots cleared more often in patients who received catheter-based treatment.

## Graphical abstract

Graphical abstract. Odds ratio (OR) comparing patients with a saddle embolus (*n* = 58) with those without (*n* = 152) for the primary composite outcome. CTPA, computed tomography pulmonary angiography; PE, pulmonary embolism; RCT, randomized controlled trial; STRATIFY, “Randomized trial of low-dose, ultrasound-assisted thrombolysis or heparin for pulmonary embolism”; USAT, ultrasound-assisted catheter-directed thrombolysis.

**Figure:**
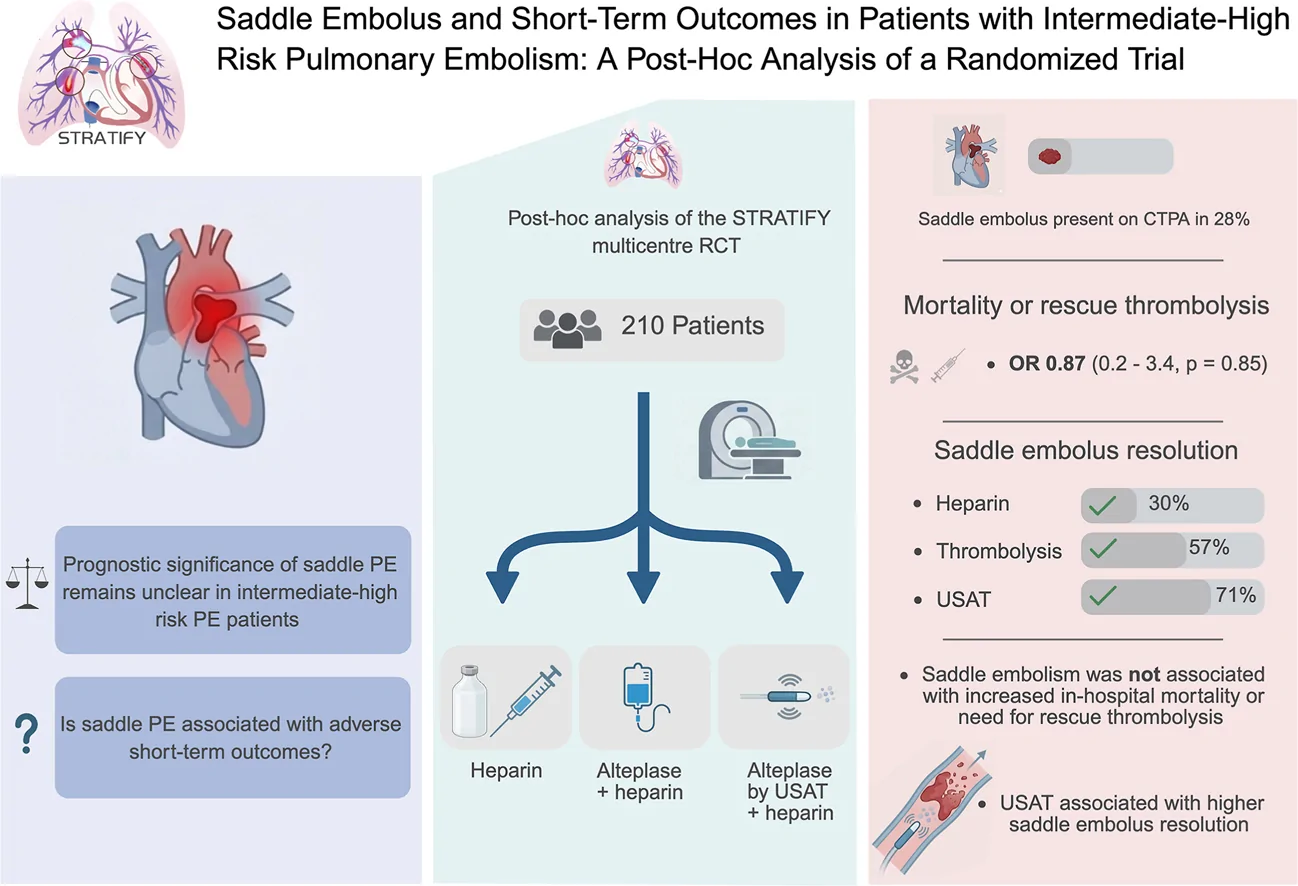

## 1 Introduction

Saddle pulmonary embolism (PE) is a radiologically distinct anatomical embolus configuration in which a thrombus straddles the bifurcation of the main pulmonary artery and is often considered a marker of disease severity [1]. Significant obstruction of the pulmonary vascular bed increases right ventricular (RV) afterload through mechanical blockage and reflex pulmonary vasoconstriction, impairing RV function and, in severe cases, leading to RV failure [2]. The reported prevalence of saddle PE ranges from 3% to 17% and occurs most frequently in patients with acute intermediate-risk PE [3]. Although estimates vary, prior studies have reported in-hospital mortality rates of up to 9.2% [[4], [5], [6]].

Intermediate-high-risk PE, defined by the presence of RV dysfunction on imaging and elevated cardiac troponin levels in the absence of circulatory shock or sustained hypotension, is associated with increased in-hospital and 30-day mortality risk [2]. However, the prognostic significance of a saddle embolus within this risk group remains uncertain. Current evidence comes from unselected PE populations, and it is unclear whether saddle embolism independently contributes to adverse outcomes such as early mortality or hemodynamic deterioration following initial stability at presentation.

We conducted a post hoc analysis of a randomized controlled trial of patients with intermediate-high-risk PE to evaluate the association between saddle embolism and short-term adverse outcomes, with a particular focus on in-hospital mortality and the need for rescue thrombolytic therapy.

## 2 Methods

This study is an exploratory post hoc analysis of data from the multicenter, randomized, open-label “Randomized trial of low-dose, ultrasound-assisted thrombolysis or heparin for PE” (STRATIFY) trial [7]. The trial investigated thrombus burden reduction with low-dose intravenous (i.v.) thrombolysis vs heparin and low-dose ultrasound-assisted catheter-directed thrombolysis (USAT) vs systemic low-dose thrombolysis. Eligible patients presented with intermediate-high-risk PE, as defined by European Society of Cardiology guidelines [2]. A total of 210 patients were randomized in a 1:1:1 ratio to 1) standard treatment with heparin (unfractionated or low-molecular-weight heparin), 2) heparin and 20 mg i.v. alteplase over 6 hours, or 3) heparin and 20 mg alteplase administered via USAT using the EKOS system (Boston Scientific) over 6 hours. The trial protocol has been published [8], along with the primary results [7]. All patients enrolled in the STRATIFY trial were eligible for inclusion in this substudy.

Demographic data were collected at trial enrollment, and clinical parameters were recorded at hospital admission. Admission electrocardiograms were reviewed for the presence of arrhythmias, an S1Q3T3 pattern, T-wave inversion in leads V1 to V3, and the presence of either complete or incomplete right bundle branch block. A bedside transthoracic echocardiogram was performed at the time of PE diagnosis by the on-call cardiologist, evaluating left ventricular (LV) ejection fraction, tricuspid regurgitation gradient (TR gradient), and the tricuspid annular plane systolic excursion (TAPSE). Admission computed tomography pulmonary angiography (CTPA) scans were independently reviewed by a study radiologist blinded to treatment allocation. RV and LV diameters were measured to calculate the RV/LV ratio, and clot burden was assessed using the refined modified Miller score (rmMS) [9]. Saddle embolus status was determined separately from the treating hospital’s original diagnostic CTPA report before randomization. Thrombus distribution on admission CTPA was summarized using the individual rmMS arterial components [9], with involvement defined as an rmMS score > 0 in the corresponding artery. Resolution of saddle embolism on follow-up CTPA (48-96 hours postrandomization) was assessed by 2 physicians, with final classification determined by consensus. Residual thrombus still straddling the pulmonary artery bifurcation on follow-up imaging was classified as persistent, even if thrombus burden had decreased relative to baseline.

The primary outcome of this post hoc analysis was a composite endpoint of rescue thrombolytic therapy for clinical deterioration and/or in-hospital death, selected to reflect the key clinical concern in managing patients with intermediate-high-risk PE.

The secondary outcome included radiographic resolution of saddle PE on follow-up CTPA, stratified by treatment modality.

Additional outcomes included differences in rmMS, oxygen saturation, and oxygen requirement at hospital admission, as well as the dyspnea index, 6-minute walking distance, D-dimer, TAPSE, and TR gradient at the 3-month follow-up.

The trial was registered at ClinicalTrials.gov (NCT04088292), and approvals were obtained from the regional ethics committee of the Capital Region of Denmark (H-18013257), the Danish Medicines Agency (2017-005075-91), and the Danish Data Protection Agency (VD-2019-52).

### 2.1 Statistical analysis

Continuous variables were reported as medians with IQRs and compared using the Wilcoxon rank-sum test, except for normally distributed variables, which were analyzed using Student’s *t*-test and reported as means with SDs. Categorical variables were presented as counts and percentages and compared using the chi-square test or Fisher’s exact test, as appropriate.

The association between saddle embolus vs no saddle embolus and the primary composite outcome of in-hospital mortality and/or escalation to rescue thrombolytic therapy was assessed using Firth’s penalized logistic regression to accommodate low event counts. The model was adjusted for sex and age, and results were reported as odds ratios (ORs) with 95% CIs. In exploratory sensitivity analyses, separate models were fitted using alternative adjustment sets: one for baseline rmMS and one for randomized treatment allocation (heparin, low-dose systemic thrombolysis, or USAT).

Resolution of saddle embolus on follow-up CTPA was compared pairwise across treatment groups (USAT, i.v. low-dose thrombolysis, and heparin) using Fisher’s exact test. ORs with 95% CIs were calculated for each comparison.

A 2-sided *P* value < .05 was considered statistically significant. No formal adjustment for multiple comparisons was performed because of the exploratory post hoc design. Statistical analyses were performed using R version 4.3.2 (R Foundation for Statistical Computing).

## 3 Results

A total of 210 patients with intermediate-high-risk PE were included, of which 58 (28%) presented with a saddle embolism. Baseline characteristics, stratified by the presence of saddle embolism on admission CTPA, are presented in Table 1. Hypertension and previous thromboembolic events were common comorbidities. No significant differences were observed in demographic characteristics, comorbidity burden, or syncope before randomization between patients with and without saddle embolism.

**Table 1:** Baseline characteristics and randomized treatment allocation of patients (*N* = 210) stratified by the presence of saddle pulmonary embolus.

Characteristic	Saddle embolus (*n* = 58)	No saddle embolus (*n* = 152)	*P* value
Female sex	31 (53)	89 (59)	.38
Age (y)	69 (61-75)	70 (62-77)	.63
BMI (kg/m^2^)	30 (27-35)	30 (26-33)	.49
Smoking	23 (40)	68 (45)	.65
Alcohol overuse	1 (2)	6 (4)	.68
Heart failure	2 (3)	0 (0)	.08
IHD	3 (5)	6 (4)	.71
Arrhythmia	1 (2)	5 (3)	1.0
Hypertension	22 (38)	66 (43)	.57
Stroke/TCI	2 (3)	5 (3)	1.0
Diabetes	9 (16)	11 (7)	.11
Asthma	5 (9)	14 (9)	1.0
COPD	1 (2)	8 (5)	.45
Renal impairment	3 (5)	7 (5)	1.0
Cancer	6 (10)	27 (18)	.21
Coagulopathy	1 (2)	8 (5)	.85
Previous DVT	7 (12)	15 (10)	.83
Previous PE	4 (7)	17 (11)	.45
Syncope	12 (21)	27 (18)	.72
Treatment allocation			.37
Heparin	22 (38)	48 (32)	
Low-dose thrombolysis	21 (36)	49 (32)	
USAT	15 (26)	56 (37)	

Data are presented as median (IQR) or *n* (%).

BMI, body mass index; COPD, chronic obstructive pulmonary disease; DVT, deep vein thrombosis; IHD, ischemic heart disease; PE, pulmonary embolism; TCI, transitory cerebral ischemia; USAT, ultrasound-assisted catheter-directed thrombolysis.

There were no significant differences between patients with and without saddle embolism in hemodynamic parameters, including heart rate, systolic blood pressure, respiratory rate, and oxygen requirements (Table 2). Biomarker levels, including lactate, D-dimer, troponin I, and troponin T, did not differ significantly between groups. Similarly, there were no significant differences in electrocardiographic signs of right-sided heart strain or cardiac function parameters. However, the total rmMS was higher in patients with saddle embolus (20 vs 19; *P* = .007), and thrombus involvement in larger, more proximal arteries was numerically more frequent. Detailed thrombus distribution by saddle embolus status is presented in the Supplementary Table. Six admission CTPA scans were technically inadequate for assessing rmMS.

**Table 2:** Clinical findings, laboratory parameters, electrocardiographic features, echocardiographic measurements, and imaging markers on admission in patients (*N* = 210) with acute pulmonary embolism, stratified according to the presence or absence of a saddle embolus.

Variable	Saddle embolus (*n* = 58)	No saddle embolus (*n* = 152)	*P* value
Heart rate (beats/min)	104 ± 19.6	101 ± 18	.24
Systolic pressure (mmHg)	135 ± 20.7	135 ± 22	.97
Saturation	96 (95-97)	95 (93-97)	.15
Oxygen need (L/min)	2 (0-4)	1 (0-4)	.90
Respiration rate	20 (18-22)	20 (18-24)	.46
Temperature (^0^C)	36.7 (36.4-37.1)	36.5 (36.2-36.9)	.01
pH	7.5 (7.4-7.5)	7.4 (7.4-7.5)	.41
pO_2_ (kPa)	8.9 (7.8-10.5)	8.7 (7.8-10.0)	.74
pCO_2_ (kPa)	4.3 (3.9-4.7)	4.3 (3.8-4.2)	.72
Lactate (mmol/L)	1.7 (1.1-2.2)	1.6 (1.2-2.6)	.66
D-dimer (FEU/L)	9.8 (6.1-19.2)	8.2 (5.5-15.0)	.22
Troponin I (ng/L)	319 (171-731)	396 (178-988)	.31
Troponin T (ng/L)	131 (76-229)	98 (44-190)	.10
Arrhythmia	4 (7)	8 (5)	.41
S1Q3T3	20 (35)	55 (36)	.95
Neg T V1-V3	23 (40)	66 (43)	.74
RBBB/iRBBB	3 (5)	21 (14)	.09
LVEF, %	60 (60-60)	60 (60-60)	.55
TAPSE (mm)	15 (13.2-16.0)	17 (13.2-18.0)	.09
TR gradient (mmHg)	40 (35-48)	42 (35-51.0)	.20
Total rmMS	20 (19-22)	19 (16-22)	.007
RV/LV ratio	1.5 ± 0.37	1.5 ± 0.34	.72

Data are presented as mean ± SD, median (IQR), or *n* (%). LVEF, TAPSE, and TR gradient were assessed by transthoracic echocardiography, and RV/LV ratio and rmMS by computed tomography pulmonary angiography. Troponin I was available in 78 patients and troponin T in 82.

FEU, fibrinogen equivalent units; iRBBB, incomplete right bundle branch block; LV, left ventricle; LVEF, left ventricular ejection fraction; Neg, negative; pO2, partial pressure of oxygen; pCO_2_, partial pressure of carbon dioxide; RBBB, right bundle branch block; rmMS, refined modified Miller score; RV, right ventricle; TAPSE, tricuspid annular plane systolic excursion; TR, tricuspid regurgitation.

The primary composite endpoint occurred in 10 patients overall. Among patients with saddle PE, 2 (3%) met the composite outcome; 1 experienced in-hospital death and 1 required rescue thrombolysis. By comparison, 8 (5%) patients without saddle PE met the composite outcome, including 4 deaths and 4 requiring rescue thrombolysis, with 1 patient experiencing both events. When assessing the primary composite outcome of in-hospital death and/or escalation to rescue thrombolysis, there was no statistically significant association between saddle PE and the odds of the composite outcome compared with nonsaddle PE (OR, 0.87; 95% CI, 0.16-3.37; *P* = .85). In exploratory sensitivity analyses, the estimate remained largely unchanged after adjustment for baseline rmMS (OR, 0.82; 95% CI, 0.15-3.22; *P* = .79) and treatment allocation (OR, 0.88; 95% CI, 0.16-3.42; *P* = .86).

Among patients with a saddle embolus, 3 did not undergo CTPA at follow-up (48-96 hours postrandomization). Of the remaining 55 patients, 14 (26%) received USAT, 21 (38%) received low-dose systemic thrombolysis, and 20 (36%) received heparin alone.

Examining the rate of saddle embolus resolution on follow-up CTPA, treatment with USAT was associated with higher odds of resolution than heparin (OR, 5.5; 95% CI, 1.06-34.92; *P* = .04). USAT also showed a numerically higher, though nonsignificant, likelihood of resolution than thrombolysis (71% vs 57%; OR, 1.84; 95% CI, 0.37-10.80; *P* = .49). Similarly, systemic thrombolysis showed a trend toward higher resolution rates than heparin (OR, 3.10; 95% CI, 0.73-13.86; *P* = .12), though this did not reach statistical significance. Overall, saddle embolus resolution was achieved in 71% of patients treated with USAT compared with 57% in the thrombolysis group and 30% in the heparin group (Figure).

**Figure:**
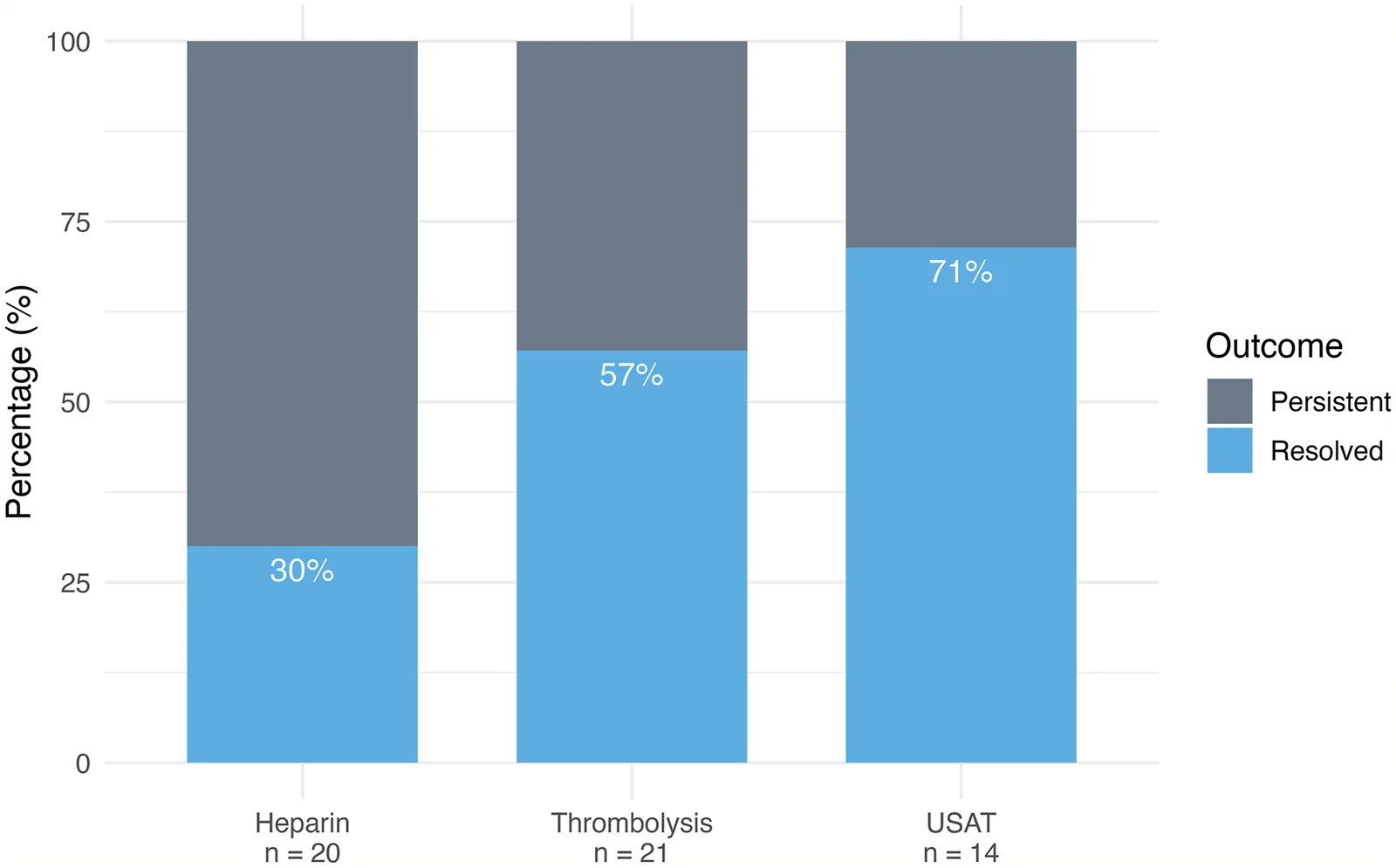
Proportion of patients with complete resolution (blue bars) and persistent saddle embolus (gray bars) on follow-up computed tomography pulmonary angiography (48-96 hours after baseline), stratified by treatment group. Among 58 patients with a baseline saddle embolus, follow-up imaging was available for 55: 20 of 22 in the heparin group, 21 of 21 in the low-dose systemic thrombolysis group, and 14 of 15 in the ultrasound-assisted catheter-directed thrombolysis (USAT) group.

At the 3-month follow-up, TAPSE was lower in patients with a saddle embolus (*n* = 181; median, 22 mm vs 25 mm; *P* = .02; Table 3). After adjustment for randomized treatment allocation, the difference was attenuated and no longer significant (adjusted mean difference, −1.47 mm; 95% CI, −3.22 to 0.29; *P* = .10). No significant difference in 6-minute walking distance was observed between patients with and without a saddle embolus (*n* = 166; median, 457 meters vs 467 meters; *P* = .67). Similarly, there were no significant differences in TR gradient (*n* = 176; median, 28.5 mmHg vs 28 mmHg; *P* = .77), dyspnea index (*n* = 181; median, 0.5 vs 0.5), D-dimer (*n* = 180; median, 0.3 vs 0.3), or rmMS (*n* = 192; median, 15 vs 15) between the 2 groups.

**Table 3:** Clinical, laboratory, and echocardiographic findings at 3-month follow-up in patients with available follow-up data (saddle embolus: 50/58; no saddle embolus: 131/152).

Variable	Saddle embolus (*n* = 50)	No saddle embolus (*n* = 131)	*P* value
Dyspnea index	0.5 (0-2)	0.5 (0-2)	.67
Six-min walking distance (m)	457 (389-525)	467 (393-554)	.67
D-dimer (FEU/L)	0.3 (0.3-0.3)	0.3 (0.3-0.4)	.85
TAPSE (mm)	22 (20-26)	25 (23-27)	.02
TR gradient (mmHg)	28.5 (24-31)	28 (24-32)	.78
Total rmMS	15 (12-19)	15 (10.5-17)	.11

Data are presented as median (IQR). Six-min walking distance *n* = 166; TR gradient *n* = 176; total rmMS *n* = 192.

FEU, fibrinogen equivalent units; rmMS, refined modified Miller score; TAPSE, tricuspid annular plane systolic excursion; TR, tricuspid regurgitation.

## 4 Discussion

In this post hoc analysis of the STRATIFY trial [7], we did not observe an association between saddle PE and early mortality or clinical deterioration compared with patients without saddle PE. Treatment with USAT was associated with a higher rate of saddle embolus resolution on follow-up CTPA at 48 to 96 hours than with heparin alone. At 3-month follow-up, no significant differences were observed in clinical or echocardiographic parameters between patients with and without a saddle embolus at admission.

Reported rates of saddle PE range from 2.6% to 17% in unselected PE populations across various risk categories [3,[10], [11], [12]]. In our study, we observed a 28% prevalence of saddle PE. This finding may be explained by including only patients with CTPA-confirmed PE who met criteria for intermediate-high risk.

Previous studies examining the prognostic significance of saddle PE report heterogeneous results. Several observational studies of unselected acute PE populations have not demonstrated differences in short-term PE-related or all-cause mortality [4,[11], [12], [13], [14], [15]]. Similarly, studies investigating the prognostic effects of embolus location—central vs peripheral—rather than morphology have also reported conflicting findings [[16], [17], [18], [19], [20], [21]].

The only meta-analysis evaluating the prognostic impact of saddle embolism reviewed 17 observational studies and 174 case reports and found a lower overall mortality rate in patients with saddle PE than in those without, likely due to publication bias [3]. In high-risk patients, saddle embolism was associated with markedly increased mortality (OR, 30; 95% CI, 4.9-182). However, the analysis included only 253 patients, mostly from case reports and smaller observational studies, introducing a substantial risk of bias due to limited data availability [3].

Similarly, a post hoc analysis of a prospective cohort study reported an association between saddle embolism and mortality in patients classified as low- or intermediate-risk [22]. The authors observed a higher prevalence of saddle embolism among intermediate-high-risk patients. Since they did not adjust for confounding variables (due to low event counts), their findings suggest that the observed increase in unadjusted mortality risk may reflect underlying risk stratification rather than saddle embolus morphology itself. Interestingly, the same authors reported an adjusted analysis showing an increased risk of hemodynamic collapse in patients with saddle embolism compared with those without. These results contrast with our findings and align with previous reports of increased risk of hemodynamic deterioration or collapse in patients with saddle PE, including a meta-analysis reporting late decompensation in 11% of patients [4,14,15,22].

It is well-established that RV dysfunction resulting from acutely elevated pulmonary artery pressure is associated with increased early mortality [2]. From a pathophysiological perspective, late deterioration or mortality may not be driven by saddle embolus morphology itself. Instead, it may depend on the degree of hemodynamic compromise from mechanical obstruction and pulmonary vasoconstriction, which increases RV afterload in patients with varying compensatory reserve. Underlying determinants may include thrombus volume, the degree of obstruction in parallel vessels, and thrombus-induced changes in pulmonary vascular tone [23]. Indeed, one study of a non–high-risk PE population found that saddle embolism was correlated with an increased risk of hemodynamic deterioration only in patients exhibiting a higher burden of peripheral artery occlusion [24]. However, embolic obstruction burden alone may not necessarily correlate with short-term mortality [18].

We found a high rate of persistent saddle embolus morphology at 48 to 96 hours (70%) among patients treated with heparin. Delayed thrombus resolution, resulting in prolonged increased afterload, may lead to progressive RV failure and precipitate late deterioration. Our findings indicate that USAT is associated with significantly higher odds of short-term saddle embolus resolution. Although saddle embolism was not independently associated with prognostic significance in our study, this observation may still be clinically relevant. Treatment modalities that promote faster clot resolution may help relieve RV strain and prevent hemodynamic deterioration in selected patients at risk of progressive RV dysfunction. Several ongoing studies are investigating these strategies [25,26].

A key strength of the present study is the inclusion of a well-characterized cohort of hemodynamically stable patients with intermediate-high-risk PE, allowing differentiation between the effects of saddle embolus morphology and immediate hemodynamic effects. Furthermore, because saddle embolus status was based on the original diagnostic CTPA reports, this classification reflects the information available to treating physicians at diagnosis, although variation in how the term was applied across reports cannot be excluded. In addition, as the study cohort was derived from the STRATIFY trial population and therefore subject to specific eligibility criteria, selection bias cannot be entirely excluded. Importantly, the present study was not prospectively powered to detect outcome differences stratified by clot morphology. Accordingly, the findings should not be interpreted as excluding a clinically relevant association. Rather, in this selected trial population, saddle morphology did not show a clear signal of markedly increased short-term mortality or deterioration, while differences in clot-resolution patterns across treatment groups warrant further study.

## 5 Conclusion

In this post hoc study of patients with intermediate-high-risk PE, we did not observe an association between saddle embolism and in-hospital mortality or the need for rescue thrombolytic therapy due to hemodynamic deterioration. However, due to the low event rate, the study had limited power to exclude clinically relevant differences in these outcomes. Among patients with saddle embolus who underwent follow-up imaging after 48 to 96 hours, treatment with USAT was associated with a higher rate of saddle embolus resolution than heparin alone.
