# Supplementary material for: Saddle embolus and short-term outcomes in patients with intermediate-high-risk pulmonary embolism: a post hoc analysis of a randomized trial
Source: Res Pract Thromb Haemost. 2026 Aug 27;10(6):106921. doi: 10.1016/j.rpth.2026.106921 (PMC13594934; doi:10.1016/j.rpth.2026.106921)
Supplement: Supplementary file 1 [file mmc1.docx]

**Supplementary Material**

| **Thrombus distribution** | **No saddle embolus** | **Saddle embolus** |
| --- | --- | --- |
| Main pulmonary artery involvement, n (%) | 8 (5%) | 24 (44%) |
| Right pulmonary artery involvement, n (%) | 25 (17%) | 33 (60%) |
| Left pulmonary artery involvement, n (%) | 28 (19%) | 32 (58%) |
|  |  |  |
| Bilateral proximal pulmonary artery involvement, n (%) | 11 (7%) | 27 (49%) |
| Right interlobar involvement, n (%) | 119 (80%) | 49 (89%) |
| Left interlobar involvement, n (%) | 104 (70%) | 44 (80%) |
|  |  |  |
| Any right-sided involvement, n (%) | 149 (100%) | 55 (100%) |
| Right upper lobe involvement, n (%) | 137 (92%) | 52 (95%) |
| Right middle lobe involvement, n (%) | 122 (82%) | 41 (75%) |
| Right lower lobe involvement, n (%) | 145 (97%) | 54 (98%) |
|  |  |  |
| Any left-sided involvement, n (%) | 146 (98%) | 54 (98%) |
| Left upper lobe/lingular involvement, n (%) | 137 (92%) | 48 (87%) |
| Left lower lobe involvement, n (%) | 140 (94%) | 54 (98%) |
|  |  |  |
| Bilateral lung involvement, n (%) | 146 (98%) | 54 (98%) |
| Number of involved territories, median [IQR] | 15 [11-19] | 16 [12-19] |
| Total rmMS, median [IQR] | 19 [16-22] | 20 [19-22] |

**Supplementary Table** Thrombus distribution on admission computed tomography pulmonary angiography according to saddle embolus status. Arterial involvement was defined as the presence of any thrombus in the corresponding artery. IQR, interquartile range; rmMS, refined modified Miller score.
